# COVID-19 and psychiatric training: Results from the efpt country surveys

**DOI:** 10.1192/j.eurpsy.2021.138

**Published:** 2021-08-13

**Authors:** A. Nobels, C. Migchels, A. Seker, C. So, P. Trančik, N. Zaja, G. Stercu

**Affiliations:** 1 Psychiatry And Medical Psychology, Ghent University Hospital, Ghent, Belgium; 2 International Centre For Reproductive Health, Ghent University, Gent, Belgium; 3 Efpt, European Federation of Psychiatric Trainees, Brussels, Belgium; 4 University Psychiatric Centre, KULeuven, Kortenberg, Belgium; 5 Child And Adolescent Psychiatry, Erciyes University Hospital, Kayseri, Turkey; 6 Faculty Of Medecine, Université de Tours, Tours, France; 7 General Adult Psychiatry, Psychiatric hospital Bohnice, Prague, Czech Republic; 8 3rd Faculty Of Medicine, Charles University, Prague, Czech Republic; 9 Nimh, National Institute of Mental Health, Klecany, Czech Republic; 10 Psychiatry, Vrapce Psychiatric Hospital, Zagreb, Croatia; 11 General Adult Psychiatry, Prof. Dr. Alexandru Obregia Clinical Hospital of Psychiatry, Bucharest, Romania

**Keywords:** online training, Education, psychotherapy, pandemic

## Abstract

**Introduction:**

Several studies link COVID-19 and the associated lockdown and social-distancing measures to adverse mental health outcomes. In order to address this increase in mental health problems, adequate training of mental health care professionals is of the utmost importance. **Objectives:** To measure the impact of the COVID-19 pandemic on psychiatric training in Europe and beyond. **Methods:** The European Federation of Psychiatric Trainees (EFPT) represents more than 20 000 trainees from over 30 European countries. Every year, country representatives, complete the ‘Country Report’, which contains detailed information on psychiatric training in every (member) country. **Results:** In July 2020, representatives of 34 European and 9 non-European countries completed the survey. In 73% of countries, psychiatric trainees were assigned to COVID-19 wards, in 43% to emergency wards. In 25% of countries, trainees did not receive any training on COVID-19 prior to their assignment. Compared to before the COVID-19 pandemic, trainees reported a decrease in clinical supervision in 65% of countries. In 51% of countries, (parts of) formal psychiatric training was cancelled. Psychotherapy training was cancelled in 25% of countries. In the majority of countries both formal and psychotherapy training were given online, however in 56% trainees experienced difficulties to attend. **Conclusions:** The COVID-19 pandemic has had an extensive impact on psychiatric training in Europe and beyond. The EFPT calls upon policy makers and supervisors to minimize the impact of COVID-19 on psychiatric training in order to provide psychiatric trainees with adequate skills to deal with the mental health consequences of the COVID-19 pandemic.

**Disclosure:**

No significant relationships.

